# Relationship between serum cystatin C and diabetic retinopathy in T2DM patients

**DOI:** 10.3389/fmed.2025.1725451

**Published:** 2025-11-17

**Authors:** Qiuqi Gui, Kunhua Jiang, Yuxin Xu

**Affiliations:** 1 Department of Ophthalmology, Second Affiliated Hospital of Anhui Medical University, Hefei, China; 2 Department of Ophthalmology, Chongming Branch, Shanghai Tenth People’s Hospital, Tongji University School of Medicine, Shanghai, China

**Keywords:** cystatin C, diabetes, diabetic retinopathy, T2DM, renal function

## Abstract

**Objective:**

Cystatin C (CysC), as a crucial and sensitive indicator for renal function, has gradually drawn attention for its role in diabetic complications. This study aims to investigate the association between serum CysC levels and diabetic retinopathy (DR).

**Method:**

This cross-sectional study enrolled 818 individuals with type 2 diabetes, including 227 DR patients and 591 patients without DR. All subjects underwent detailed clinical evaluations, including blood glucose, lipid, renal function indicators, and fundus examinations. Logistic regression analyses were applied to assess the correlation between CysC and DR.

**Results:**

The serum CysC levels in DR patients was significantly higher than those of the controls (*p* < 0.001). Besides, CysC was negatively correlated with fasting glucose (*r* = −0.080), TC (*r* = −0.090), HDL-C (*r* = −0.107), and albumin (*r* = −0.222) (all *p* < 0.05). Compared to the 1st tertile of CysC, the prevalence of DR was increased in the 3rd CysC tertile (OR = 2.14, 95%CI: 1.20–3.82, *p* = 0.01). This association was more obvious in patients with a long duration of diabetes exceeding 10 years or in non-elderly patients.

**Conclusion:**

Patients with higher serum CysC levels have an elevated risk of DR in the T2DM population. Future large-scale studies should explore the potential mechanism of CysC in DR and evaluate its potential as a therapeutic target.

## 1 Introduction

Type 2 diabetes mellitus (T2DM) is currently one of the most severe and prevalent chronic diseases. Diabetic retinopathy (DR), a major microvascular complication of T2DM, is the leading cause of vision loss among working-age populations worldwide (1). Epidemiological data shows that in 2020, over 103 million diabetic patients worldwide suffered from DR. It is estimated that by 2045, this number will increase to 160 million (2). Compared to other major causes of blindness, DR was the only disease whose age-standardized prevalence did not decline during the period from 1990 to 2020 (3). In addition to affecting vision, DR is linked to heightened risks of depression and cardiovascular and cerebrovascular diseases (4–7). Therefore, identifying modifiable risk factors for DR at an early stage is essential for its prevention and treatment.

Cystatin C (CysC), a sensitive and effective indicator for renal function, is a lysosomal cysteine proteinases inhibitor generated by all nucleated cells (8). High levels of CysC are closely related to oxidative stress, inflammation, and endothelial dysfunction. Additionally, an increase in CysC concentration may enhance the suppression of cysteine proteases, potentially contributing to the onset and progression of microvascular and macrovascular diseases (3). In addition to being associated with kidney diseases, patients with higher CysC levels also have a greater risk of cardiovascular disease, cancer, and all-cause mortality (9).

Recent studies have indicated a strong association between CysC and the risk of diabetes and its complications (10). A retrospective study showed that compared with the control group, the levels of CysC in patients with diabetes remained unchanged, while the levels of CysC in patients with DR increased (11). He et al. (12) reported that the serum CysC level was correlated with the severity of DR and can predict DR that poses a threat to vision. Besides, Kim et al. (13) found that the serum CysC level was independently associated with the prevalence of DR and coronary heart disease in a group of Korean T2DM patients without nephropathy. Moreover, several studies have shown that CysC can serve as a specific biomarker for patients with DR (11, 14, 15). Xiong et al. (16) revealed that elevated CysC levels is tightly correlated with microvascular rarefaction in optic disc and macular regions, as well as diminished retinal neural layers among diabetic subjects. Besides, CysC was a crucial and independent predictor of peripheral arterial stiffness among T2DM subjects with chronic kidney disease (17). However, prior studies involved participants with a broad spectrum of kidney functions, which means the link between CysC and DR might be influenced by factors related to kidney issues. Therefore, the aim of this study was to explore the relationship between higher serum CysC levels and the risk of DR in T2DM patients with normal renal function.

## 2 Methods

### 2.1 Study participants

This cross-sectional study included 818 subjects in the Department of Endocrinology and Ophthalmology of Chongming Branch, Shanghai Tenth People’s Hospital, Tongji University School of Medicine between March 2020 and November 2024. Patients aged ≥18 years diagnosed with T2DM according to Chinese Diabetes Society criteria were included. Exclusion criteria were as follows: non-T2DM patients; patients with hyperthyroidism, patients with incomplete information, patients with renal dysfunction (serum creatine >1.3 mg/dL, urine albumin excretion rate ≥30 mg/day, or estimated glomerular filtration rate (eGFR) <60 mL/min/1.73m^2^); patients with diabetic acute complications, and severe or recurrent hypoglycemic events; patients with malignant tumors, psychiatric disorders, infections, or other organ failures; and patients with glaucoma, previous vitreous surgery, or cataract. The study was approved by the Ethics Committee of the Chongming Branch, Shanghai Tenth People’s Hospital (Approval Number: SHSYCM-IEC-1.0/25-YF/04).

### 2.2 Data collection

Clinical data were obtained from the electronic medical records, including age, sex, height, weight, smoking and drinking status, duration of diabetes, medical history and usage of insulin. Body mass index (BMI) was calculated as weight divided by height squared. Smoking refers to a self-reported history of smoking or currently smoking. Hypertension refers to a resting blood pressure of 140 mmHg systolic or 90 mmHg diastolic or higher on repeated measurements, or the use of antihypertensive medications (18). Laboratory tests include CysC, glycated hemoglobin (HbA1c), fasting blood glucose (FBG), low-density lipoprotein cholesterol (LDL-C), triglycerides (TG), high-density lipoprotein cholesterol (HDL-C), total cholesterol (TC), albumin, and serum creatinine were measured using an automatic biochemical analyzer in the hospital’s medical laboratory according to the routine procedures.

All participants underwent a standardized clinical examination. DR was evaluated by trained ophthalmologists according to the presence of one or more of the following indicators: retinal microvascular abnormalities, hard exudates, microaneurysm formation, venous beading, retinal neovascularization, intraretinal hemorrhage, fibrous proliferation, cotton-wool spots, and macular edema. The control group had no manifestations or clinical history of DR, and imaging studies confirmed the absence of DR.

### 2.3 Statistical analysis

Unless stated otherwise, quantitative data were shown as mean ± standard, while qualitative variables were expressed as numbers and percentages. Group differences were assessed using Student’s *t*-test or chi-square tests. Spearman correlation analysis was performed to determine the correlation between CysC and metabolic indicators. The relationship between CysC and DR prevalence was analyzed using three multivariable logistic regression models: (1) Model 1: unadjusted; (2) Model 2: adjusted for age, sex, smoking, drinking, and hypertension; (3) Model 3: further adjustments for other parameters includes BMI, diabetic duration, insulin therapy, HbA1c, albumin, and serum creatinine. Data analyses were conducted using SPSS 26.0 and GraphPad Prism 9.0. Statistical significance was set as *p* < 0.05.

## 3 Results

### 3.1 Baseline characteristics of the cohort

A total of 818 participants were included, of whom 591 were controls and 227 had DR. The average age was 61.5 ± 10.9 years, 533 (63.8%) were male, and the mean CysC levels were 0.89 ± 0.21 mg/L. The percentage of subjects with hypertension and usage of insulin in DR subjects were significantly higher than that of in controls (*p* < 0.05). They were also more prone to having a higher index of BMI and a longer duration of diabetes. Moreover, DR subjects exhibited higher levels of HbA1c, serum creatinine, and CysC, but lower albumin levels (Table 1).

**Table 1:** Baseline characteristics of the cohort.

Variables	All (*n* = 818)	CON (*n* = 591)	DR (*n* = 227)	*p*
Age, years	61.5 ± 10.9	61.5 ± 11.3	61.6 ± 10.0	0.983
Male, %	533 (63.8)	383 (64.8)	139 (61.2)	0.341
Smoking, %	272 (33.3)	202 (34.2)	70 (31.0)	0.376
Drinking, %	139 (17.0)	104 (17.6)	35 (15.5)	0.467
Hypertension, %	486 (59.4)	336 (56.7)	151 (66.5)	0.010
BMI, kg/m^2^	24.3 ± 3.3	24.1 ± 3.3	24.7 ± 3.4	0.025
Diabetic duration, years	11.1 ± 7.3	10.1 ± 7.4	13.8 ± 6.5	<0.001
Insulin therapy, %	436 (53.3)	279 (47.2)	157 (69.2)	<0.001
Fasting glucose, mmol/L	7.7 ± 2.4	7.6 ± 2.3	7.9 ± 2.7	0.108
HbA1c, %	8.9 ± 2.2	8.7 ± 2.2	9.2 ± 1.9	0.012
TG, mg/dL	170.1 ± 134.2	169.2 ± 135.1	172.7 ± 132.1	0.738
TC, mg/dL	170.1 ± 42.8	169.1 ± 40.5	172.8 ± 48.4	0.308
HDL-C, mg/dL	41.8 ± 11.2	41.8 ± 11.1	41.9 ± 11.6	0.872
LDL-C, mg/dL	108.2 ± 38.8	107.2 ± 36.6	110.9 ± 44.1	0.254
Albumin, g/L	38.7 ± 3.8	39.0 ± 3.7	37.7 ± 3.6	<0.001
Serum creatinine, mg/dL	0.81 ± 0.20	0.79 ± 0.19	0.84 ± 0.22	0.008
eGFR, mL/min/1.73m^2^	101.4 ± 60.46	103.7 ± 69.0	95.5 ± 27.2	0.083
CysC, mg/L	0.89 ± 0.21	0.86 ± 0.20	0.96 ± 0.23	<0.001

The values represent the mean ± standard deviation.

BMI, body mass index; HbA1c, glycated hemoglobin; TG, triglycerides; TC, total cholesterol; LDL-C, low-density lipoprotein cholesterol; HDL-C, high-density lipoprotein cholesterol; eGFR, estimated glomerular filtration rates; CysC, cystatin C.

### 3.2 Correlation between CysC and metabolic indicators

**Figure 1:**
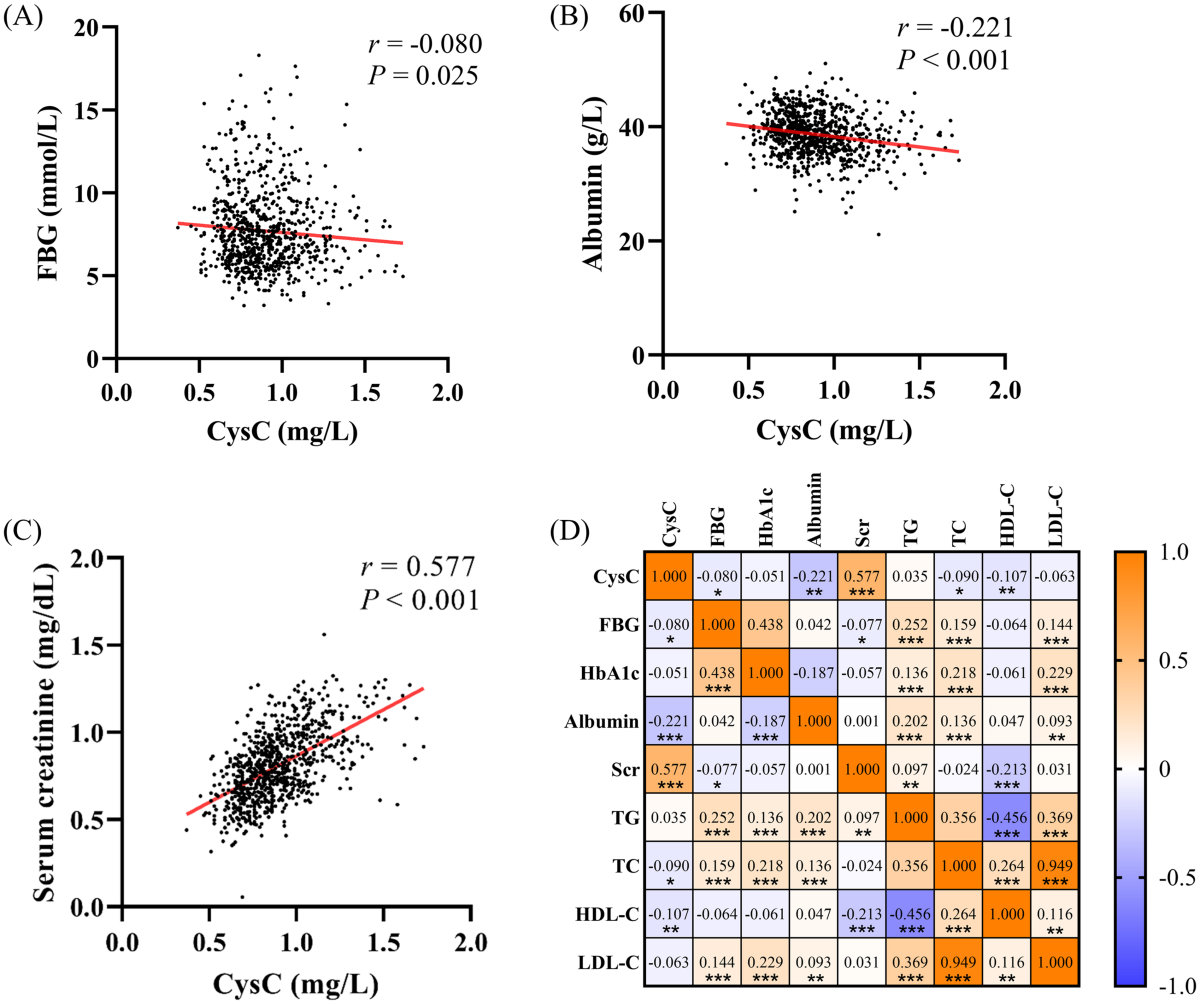
Association between serum CysC levels and metabolic indicators. **(A)** The correlation between CysC and FBG. **(B)** The correlation between CysC and albumin. **(C)** The correlation between CysC and serum creatinine. **(D)** Correlation results displayed as heat maps.

The CysC values negatively correlated with FBG (*r* = −0.080, Figure 1A), TC (*r* = −0.090), HDL-C (*r* = −0.107), and albumin (*r* = −0.222, Figure 1B) (all *p* < 0.05). Moreover, there was a positive correlation between CysC and serum creatinine (*r* = 0.577, *p* < 0.001, Figure 1C) (Table 2 and Figure 1D).

**Table 2:** Correlation of CysC and metabolic indicators.

Variables	*r*	*p*-value
Fasting glucose	−0.080	0.023
HbA1c	−0.051	0.169
TG	0.035	0.319
TC	−0.090	0.010
HDL-C	−0.107	0.002
LDL-C	−0.063	0.073
Albumin	−0.222	<0.001
Serum creatinine	0.577	<0.001

### 3.3 Association between the CysC and DR

**Figure 2:**
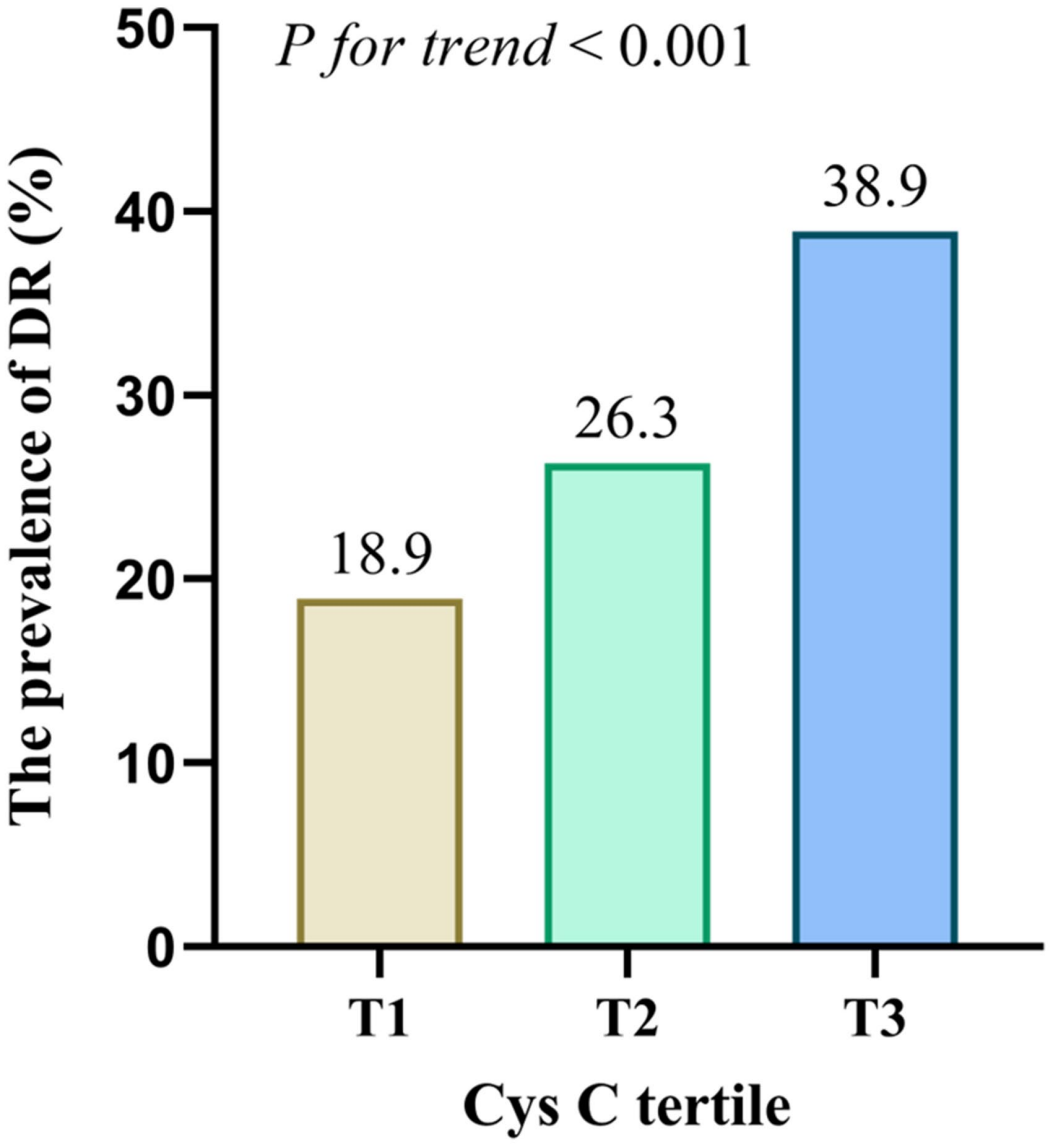
Prevalence of DR across the CysC tertiles. The prevalence of DR was increased in T2 and T3 groups compared to T1 group (*p* < 0.001).

Participants were categorized into three groups based on the tertiles of CysC levels: T1 (≤0.78 mg/L), T2 (0.79–0.95 mg/L), and T3 (≥0.96 mg/L). The percentage of DR prevalence were 18.9, 26.3, and 38.9% in the T1, T2, and T3 group, respectively (*p*_for trend_ < 0.001, Figure 2). To further determine whether CysC was promising as a predictor of DR, three multivariable logistic regression analysis were performed. When taking the T1 tertile of CysC as a reference, the prevalence of DR was increased in another two groups, with the odds ratio (OR) [95% confidence interval (CI)] were 1.53 (1.03–2.29) for T2 and 2.74 (1.86–4.03) for T3 (all *p* < 0.05, Table 3). Besides, the association remains statistically significant after the adjustment of age, sex, smoking, and drinking, and hypertension (Model 2). Moreover, a slightly higher risk was observed in the highest CysC tertile after adjustments for Model 2 variables and BMI, diabetic duration, insulin therapy, HbA1c, albumin, and serum creatinine (OR = 2.14, 95%CI: 1.20–3.82, *p* = 0.01).

**Table 3:** Multivariate logistic regression analysis of CysC for DR.

Variables	Model 1		Model 2		Model 3	
	OR (95% CI)	*p*-value	OR (95% CI)	*p*-value	OR (95% CI)	*p*-value
T1	Reference		Reference		Reference	
T2	1.53 (1.03–2.29)	0.037	1.71 (1.13–2.60)	0.011	1.53 (0.95–2.49)	0.083
T3	2.74 (1.86–4.03)	<0.001	3.35 (2.19–5.12)	<0.001	2.14 (1.20–3.82)	0.010
*p* for trend			<0.001		0.008	

Model 1: unadjusted.

Model 2: adjusted for age, sex, smoking, drinking, and hypertension.

Model 3: adjusted for age, sex, smoking, drinking, hypertension, BMI, diabetic duration, insulin therapy, HbA1c, albumin, and serum creatinine.

### 3.4 Sensitivity analysis

**Figure 3:**
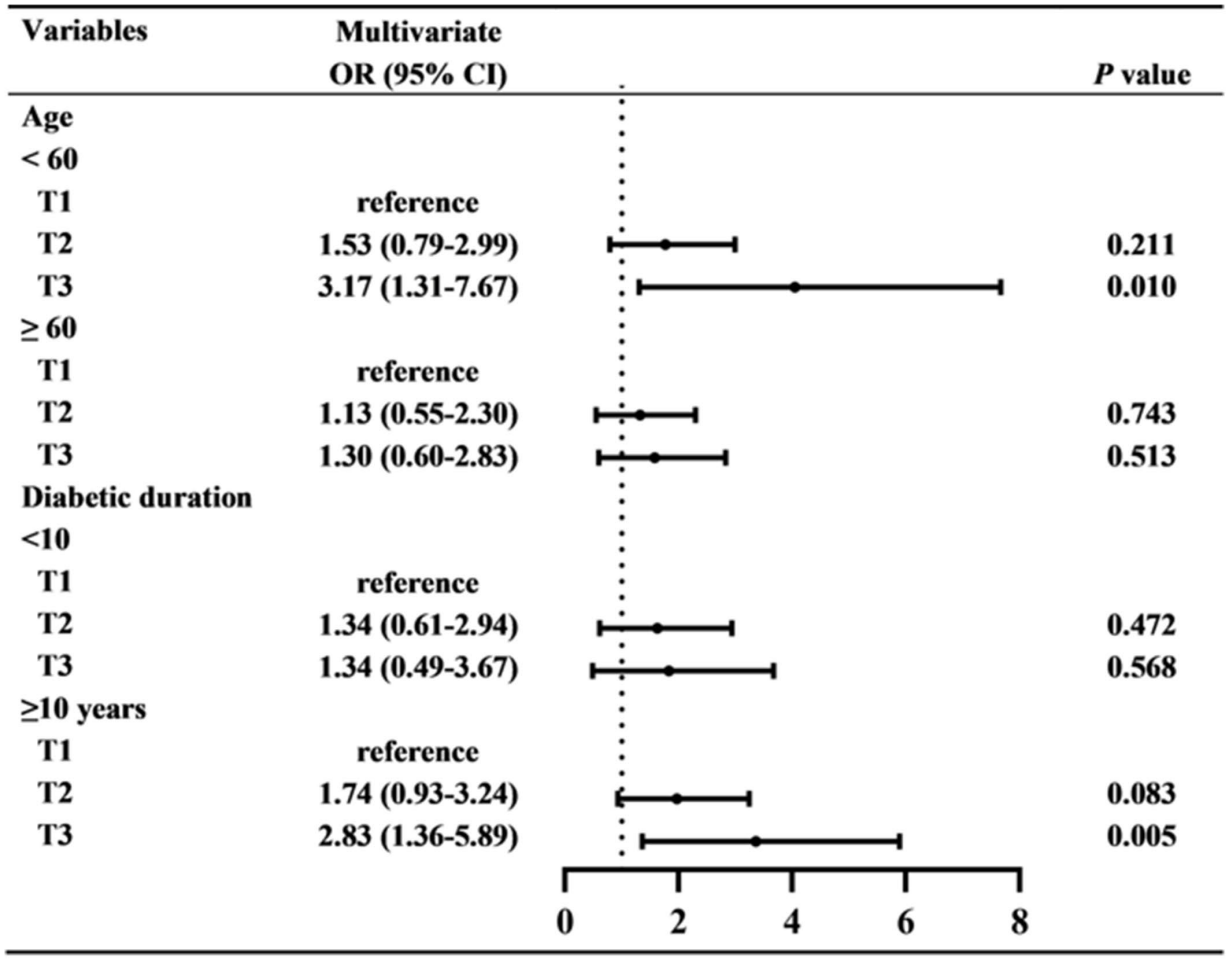
The relationship between CysC and DR in subgroups. Multivariate logistic regression analyses were used in subgroups based on age (<60 OR ≥60 years), and diabetic duration (<10 OR ≥10 years).

Subsequently, subgroup analyses were used to further investigate the relationship between CysC and DR (Figure 3). It has been found that higher serum CysC values were related to the prevalence of DR in patients aged below 60 years (OR = 3.17, 95%CI: 1.31–7.67, *p* = 0.010) and diabetic duration more than 10 years (OR = 2.83, 95%CI: 1.36–5.89, *p* = 0.005). However, for patients aged over 60 years and diabetic duration below 10 years, higher CysC did not indicate a greater risk of DR.

## 4 Discussion

DR is a severe microvascular complication of diabetes mellitus, posing a significant threat to the vision of patients. The pathogenesis of DR is intricate, with multiple contributing factors and mechanisms, including oxidative stress, inflammation, metabolic disorders caused by long-term hyperglycemia, insulin resistance, and endothelial dysfunction (19). It is of great clinical significance to identify efficient and sensitive biomarkers for DR that can be used for early diagnosis, disease progression monitoring, and prognosis assessment. This study revealed that the value of cystatin C was correlated with FBG, TC, HDL-C, and albumin, but not with HbA1c, mainly consistent with previous findings (20). Another study found a positive correlation between CysC and HbA1c in in Korean Adults (21). Besides, Stankute et al. (22) reported that CysC was negatively associated with HbA1c and HDL. Moreover, the present study determined that higher serum CysC levels were associated with the risk of DR in T2DM patients with normal renal function. As a sensitive indicator for evaluating renal function, CysC testing is convenient and fast, which could be used as an effective and simple tool for DR risk assessment in clinical practice.

The positive correlations among CysC and cardiovascular outcomes and mortality have been confirmed in diverse populations, including general population with normal eGFR (23), chronic kidney disease patients (24), obstructive sleep apnea subjects (25), patients with metabolic syndrome (9), and patients with coronary heart disease (26). Furthermore, CysC has been pinpointed as a potential indicator for various diabetic complications, including early renal damage (22), peripheral artery disease (27), diabetic foot ulcers (28), diabetic peripheral neuropathy (29), and DR (11). A cross-sectional study among the Indian population revealed that CysC may emerged as a biomarker for screening sight-threatening DR (30). Our results were mainly consistent with previous studies, which have shown that CysC levels was markedly elevated in DR patients compared to the controls (11–13, 15, 30). He et al. (12) reported that the serum CysC level was correlated with the severity of DR and can predict DR that poses a threat to vision. Besides, Kim et al. (13) found that the serum CysC level was independently associated with the prevalence of DR and coronary heart disease in a group of Korean T2DM patients without nephropathy. However, the subjects recruited in previous studies had a broad spectrum of renal functions, the relationship between CysC and DR might be influenced by renal dysfunction-related confounding factors. The present study excluded patients with nephropathy to eliminate the confounding effect of declining renal function, which helps to make our results more convincing. In addition, this study found that CysC was correlated with fasting glucose, TC, HDL-C, and albumin. These factors have also been proven to be associated with DR prevalence. It has been reported that CysC was associated with dyslipidemia (20). A population-based study includes individuals without chronic kidney disease revealed that for every one standard deviation increase in serum CysC levels, the risk of dyslipidemia increased by 22% (31).

The fundamental mechanism for the relationship between CysC and DR has not been fully elucidated. One of the key mechanisms may be that CysC causes chronic inflammatory responses. CysC is mainly synthesized in the retinal pigment epithelium and secreted from the basal side (32). Multiple studies have revealed a significant linear correlation between CysC and the levels of classic inflammatory markers, such as C-reactive protein (CRP), high-sensitivity CRP, and IL6 (32–35). Increased CRP levels are involved in the pathogenesis of DR. In the streptozotocin-induced diabetic rat models, overexpression of human CRP protein exacerbates diabetic-induced retinal leukocyte stasis and degranulated capillary formation. In retinal cell lines, human CRP protein treatment induces overexpression of reactive oxygen species and cell death. Moreover, CRP induced the upregulation of pro-inflammatory, pro-angiogenic, and pro-oxidative parameters by CD32 and NF-κB signaling pathways (36). There was a significant association between serum levels of CysC and oxidative stress index (37). CysC has been proven to be involved in macular degeneration, neovascularization, vascular integrity, inflammation, and neuronal degeneration (12). The pathophysiological alternations of DR include inflammation, optic neuropathy, macular edema, oxidative stress, and retinal neovascularization (38). The shared pathways between CysC and DR might partially clarify their strong connection.

Vascular endothelial growth factor (VEGF) participates in the regulation of endothelial cell proliferation, assembly, maintenance and survival (39). However, in the diabetic state, VEGF expression is upregulated, leading to deviation from its normal physiological function and triggering multiple pathological manifestations, such as enhanced endothelial permeability, angiogenesis, and activation of inflammatory mediators, which are essential in the development and pathophysiology of DR. Anti-VEGF antagonists are widely used for treating ocular diseases through intravitreal injection (40). A study on patients with systemic lupus erythematosus found that CysC was positively correlated with VEGF (41). Additionally, CysC participates in the regulation of VEGF secretion in the neurovascular units (42). The evidence indicates that VEGF might serve as a regulatory mechanism connecting CysC and DR. One of the typical characteristics of DR is endothelial cell dysfunction (43). The low reactive hyperemia index indicates more severe endothelial dysfunction. Kreslová et al. (44) found that CysC was independently correlated with a decreased reactive hyperemia index. After adjusting for confounding factors, increased level of CysC was an independent predictor for endothelial dysfunction.

Another important finding of this study is the presence of an age-related difference in the association between serum CysC levels and DR. Higher CysC levels were prominently correlated with DR in subjects aged <60 years, but not in older individuals. A study found that after the age of 50–60, the level of CysC begins to increase significantly. This upward trend may attributed to the gradual decline in renal function, as CysC is mainly cleared through filtration by the kidneys, and reduced renal function leads to its accumulation in the blood (45). Additionally, age is one of the essential risk factors for DR. The relationship between CysC and DR may be obscured by the influence of advanced age.

Although the study excluded patients with renal dysfunction, there are still some limitations. Firstly, the sample size of this study is relatively small, so it may not accurately represent the study population. Secondly, this study employed a cross-sectional design, which means it cannot establish a causal relationship between CysC and the risk of DR. Thirdly, the present study did not analyze the correlation between CysC and VEGF and inflammatory indicators. Further research is still needed to understand their roles in the relationship between CysC and DR. Moreover, more in-depth, prospective, and large-scale studies are required to elucidate the relationship between cystatin C and the distinct severity stages of DR, including non-proliferative DR (varying from mild to severe) and proliferative DR.

In conclusion, higher serum CysC levels in T2DM patients with normal renal function are closely related to DR. CysC may help identify high-risk individuals with DR among patients without kidney disease. Large-scale studies should determine the potential mechanism of the link between CysC and DR and investigate whether CysC-targeting therapy can halt disease initiation and progression.

## Data Availability

The raw data supporting the conclusions of this article will be made available by the authors, without undue reservation.
